# Communication of CD8^+^ T cells with mononuclear phagocytes in multiple sclerosis

**DOI:** 10.1002/acn3.783

**Published:** 2019-06-14

**Authors:** Matea Konjevic Sabolek, Kathrin Held, Eduardo Beltrán, Anna G. Niedl, Edgar Meinl, Reinhard Hohlfeld, Hans Lassmann, Klaus Dornmair

**Affiliations:** ^1^ Institute of Clinical Neuroimmunology Biomedical Center and Hospital of the Ludwig‐Maximilians‐University Munich D‐82152 Munich Germany; ^2^ Munich Cluster for Systems Neurology (SyNergy) D‐81377 Munich Germany; ^3^ Center for Brain Research Medical University of Vienna Spitalgasse 4 A‐1090 Vienna Austria

## Abstract

**Objective:**

CD8^+^ T cells are the most prevailing lymphocyte population in inflammatory lesions of patients with multiple sclerosis (MS) but it is not even known whether they are merely passive bystanders or actively communicate with other cells in the brain. To identify their potential interaction partners, we analyzed CD8^+^ T cells that contained vectorially oriented cytotoxic granules and analyzed the areas to which the granules pointed.

**Methods:**

We stained cryo‐sections of active MS lesions of an index patient with antibodies to CD8 and perforin, searched for vectorially oriented perforin granules, and isolated target areas opposing the granules and control areas by laser‐microdissection. From both areas, we analyzed cell‐type specific transcripts by next‐generation sequencing. In parallel, we stained samples from the index‐patient and other patients by four‐color immunohistochemistry (IHC).

**Results:**

We found transcripts of the mononuclear phagocyte (MP) specific markers CD163 and CD11b only in the microdissected target areas but not in control areas. We validated the finding that MPs are communication partners of CD8^+^ T cells in MS lesions by classical IHC in samples from the index‐patient and other patients with acute and progressive MS and other inflammatory neurological diseases.

**Interpretation:**

Because CD163 and CD11b are specifically expressed in MPs, our findings suggest that CD8^+^ T cells communicate with local MPs. Although it is still unclear if these interactions lead to killing of the communication partners by CD8^+^ T cells, our data underline that CD8^+^ T cells play an active role in the pathogenesis of MS.

## Introduction

Lymphocyte infiltrations into the central nervous system (CNS) are observed in all patients with acute and chronic forms of multiple sclerosis (MS).^1^, ^2^, ^3^, ^4^ Decades ago it was found that the most prevalent lymphocyte population in MS lesions are CD8^+^ T cells^5^, ^6^ and this was recently confirmed in much more detail in large cohorts.^7^, ^8^, ^9^ CD8^+^ T cells are present at perivascular sites and in the parenchyma of all patients regardless of the disease course,^3^ are often clonally expanded,^8^, ^10^, ^11^, ^12^ may accumulate in clusters,^13^ and distinct expanded clones may be found in different lesions and also in normal appearing white matter.^7^, ^12^ Many of the infiltrating T cell clones show the antigen‐experienced, tissue‐resident, memory phenotype.^7^, ^9^, ^13^ This suggests that they are involved in the pathogenesis, but so far it is unknown with which target cells they communicate within the lesions.

Specific interactions of individual cells in tightly packed tissues are experimentally difficult to investigate because each cell is usually surrounded by many other cells. Often the neighboring cells are of different type, and in inflamed tissue the situation becomes even more complicated because CNS tissue is invaded by additional types of cells. In MS, these challenges cumulate: many different cell types are tightly packed in the CNS and some of them have irregular shapes and long extensions (e.g., neurons, astrocytes, oligodendrocytes).^3^, ^14^ Furthermore, many brain‐resident cells are of the mononuclear phagocyte (MP) lineage, which contains many subtypes of high plasticity (e.g., macrophages, microglia, monocytes). These cells may switch their phenotype rapidly in situ depending on changes of the local environment.^15^, ^16^, ^17^

To identify the communication partners and putative target cells of the CNS‐invasive CD8^+^ T cells, we made use of the “killing machinery” of cytotoxic CD8^+^ T cells. After activation by their antigen‐specific T‐cell receptor they rearrange their cytoskeleton and form an immunological synapse, where cytotoxic granules are oriented toward their target cell.^18^ Granules contain perforin, which may form channels in the membrane of the target cell, as well as several granzymes which induce apoptosis. This process is strictly vectorial, that is, it is exclusively directed toward the target cell. By contrast, resting T cells store their granules isotropically distributed in the cytosol. Vectorial orientation of cytotoxic granules in CD8^+^ T cells has been visualized in autoimmune myositis and Rasmussen′s encephalitis by immunohistochemistry (IHC)^19^, ^20^ providing evidence for direct interaction with their respective targets. Here we detected vectorial perforin orientation in a small number of CD8^+^ T cells in active lesions of an index patient with MS. Using laser capture microdissection (LCM), we then “blindly” isolated the areas to which the granules pointed, that is, where the target cells were presumably localized. Analysis of cell‐type specific transcripts by next generation sequencing (NGS) revealed MPs as communication partners of the CD8^+^ T cells. We validated this result by IHC in the index patient, and in a larger cohort of additional patients with acute or progressive MS. Our unbiased approach will be applicable to many different conditions and diseases where cytotoxic T cells are involved and the target cell is unknown.

## Patients and Methods

### Clinical samples, case selection, and neuropathology

**Figure 1:**
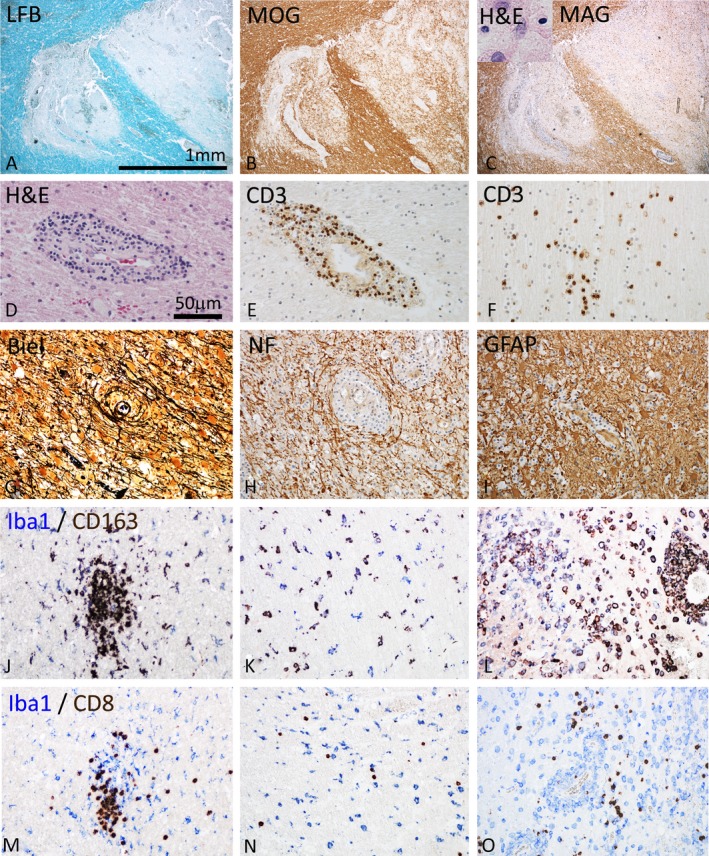
Pathological features of inflammatory demyelinating brain lesions in index patient A. (A–C) Focal plaques of demyelination with loss of myelin in Luxol fast blue myelin stain (A), partial preservation of myelin oligodendrocyte glycoprotein (MOG) reactivity in particular in initial lesion stages (B), but complete loss of myelin associated glycoprotein (MAG; C) and apoptotic nuclear changes of oligodendrocytes (C, insert). Magnification bar is applicable for (A–C). (D–F) The demyelinated lesions occur on the background of profound brain inflammation, reflected by perivascular cuffs seen in hematoxylin/eosin staining (D). The inflammatory reaction dominantly contains CD3^+^ T‐lymphocytes, which are present within the perivascular cuffs (E) and diffusely dispersed in the lesion parenchyme (F). (G–I) Characteristic features of the lesions are preservation of axons within the lesions, shown by Bielschowsky silver impregnation (Biel) (G) and immunohistochemistry for neurofilament protein (NF) (H) and the pronounced astrocytic gliosis, seen after staining for glial fibrillary acidic protein (GFAP, I). (J–L) Double staining for the pan microglia/macrophage marker Iba‐1 (blue) and the macrophage activation antigen CD163 (brown) in the periplaque white matter with microglia clusters (J), in initial lesions with diffuse microglia activation (K) and in the demyelinated lesion center with profound macrophages infiltration and activation (L). Only a subset of microglia/macrophages also expresses the activation marker CD163. (M–O) Double staining for the pan microglia/macrophage marker Iba 1 with CD8 in the periplaque white matter (M), the initial lesion (N), and the active demyelinated lesion (O). In all lesion stages CD8 cells can be seen, which are in close apposition with microglia/macrophages. Magnification bar in (D) applicable for (D–O).

Patient A^10^ presented with recurrent episodes of left sided hemianopsia and on the basis of a suspected glioblastoma the lesion located in the right temporo‐occipital white matter was surgically resected and tissue blocks were embedded in paraffin and snap frozen. Neuropathological classification was performed on the paraffin embedded mirror blocks and the diagnosis of a highly active MS‐like inflammatory demyelinating lesion was established by the presence of MS typical inflammatory reaction and plaque‐like primary demyelination with preservation of axons (Fig. 1A–I). Although such a case is atypical for MS, due to the very severe and fulminant disease course and the very large actively demyelinating lesions, such patients develop classical MS in follow‐up and the pathology of active lesions is closely similar to that seen in comparably staged lesions in patients with relapsing MS.^21^ The big biopsy specimen was heterogeneous, containing areas of normal‐appearing and periplaque white matter, areas of initial (prephagocytic) demyelinating lesions according to Ref., 22 and active demyelinated lesions with abundant macrophages, containing early myelin degradation products.^23^ The regions of interest studied here included periplaque white matter (Fig. 1J and M), as well as initial (prephagocytic) lesions (Fig. 1K and N). In addition we included areas of active demyelinated plaques, which showed a profound perivascular and diffuse infiltration of CD8^+^ T‐cells, complete myelin destruction and the abundance of activated macrophages with early myelin degradation products (Fig. 1L and O). CD163 was highly expressed in a subset of MPs at sites of active demyelination (Fig. 1J–L). Classification of lesion stages was performed as outlined in detail before.^21^

To ensure that the proper lesion areas were selected for microdissection, snap frozen mirror sections were stained for myelin and microglia/macrophages and the matching areas of periplaque white matter as well as initial and active demyelination were identified, before the subsequent sections were stained for CD8^+^ T‐cells, or used for triple or quadruple immunohistochemical staining. To validate the findings regarding T‐cell/target‐cell interaction, we selected out of a much larger sample of MS and control brain autopsies, described in detail in an earlier report on the inflammatory response in the MS brain,^9^ cases with highly active demyelination lesions and pronounced CD8^+^ T‐cell dominated inflammatory reaction (see Table 1).

**Table 1:** Cell numbers and pairs of CD8^+^ cells in MS patients and controls

Patient	Diagnosis	Age	Average number of cells/mm^2^						Pairs		Perforin expression
			CD8		CD163		CD11b				
			Parench.	Periv.	Parench.	Periv.	Parench.	Periv.	CD8/CD11b/CD163	CD163/CD8/perforin	
A10a^a^	AMS	49	95	51	379	120	310	75	Yes	Yes	+/++
A12b^a^	AMS	49	87	8	187	15	257.5	31	Yes	Yes	+/++
A11a^a^	AMS	49	56	14	0	4	169	9.5	No	No	+
A11b^a^	AMS	49	30.5	2.5	0	2	95	6	No	No	+
MS11^b^	AMS	78	91	12	400	55	24	2	Yes	Yes	+/++
MS10^b^	AMS	69	2	10	410	60	51	14.5	Yes	Yes	+
MS3^b^	AMS	45	2.5	60	71	60	110	34	Yes	No	−/+
MS4^b^	AMS	45	1	6	0	10	31	45	No	No	ND
MS2^b^	AMS	35	1	14	51	10	7	61	Yes	No	−
MS25^b^	SPMS	48	3	20	360	40	14	16	Yes	Yes	+
MS22^b^	SPMS	41	1	35	112	90	35.5	54.5	Yes	No	−/+
MS1854^c^	PPMS	42	2	16	5	8	4	2	No	No	+
NMO 499^d^	NMO	20	6.5	20	276	35	308	48	Yes	No	+
Y304/12/52	Rasmussen	9	25	10	0	40	15	5	No	No	−/+

Patients, diagnoses, and ages are listed in columns 1–3. Four different tissue blocks (10a, 12b, 11a, and 11b) from a biopsy sample from patient A were used. Columns 4–9 list median cell numbers/mm^2^ for CD8^+^ (columns 4 and 5) CD163^+^ cells (columns 6 and 7) and CD11b+ cells (columns 8 and 9) detected in the parenchyma (Parench.) (columns 4, 6, and 8) and perivascular (Periv.) space (columns 5, 7, and 9). Cell numbers/mm^2^ were determined in areas with high inflammatory activity. Column 10 lists whether or not CD8^+^ cells could be detected in direct contact with CD11b^+^CD163^+^ cells. In column 11 we list whether or not CD8^+^ cells in direct contact with CD163^+^ cells expressed perforin. In column 12 we give a semi‐quantitative estimation of the perforin expression level in CD8^+^ cells irrespective of whether they contacted CD163^+^ cells or not: ND = not done; “−” = not present; “−/+” = weak expression or <10% CD8^+^ cells; “+” = moderate expression or in 10–40% of CD8^+^ cells; “++” = strong expression or in more than 40% CD8^+^ cells; AMS, acute multiple sclerosis.

a Patient described in Ref. 10.

b Patients described in Ref. 9.

c Patient MS1854 represents "MS4" in Ref. 12.

d Patient described in Ref. 39.

The studies on human samples were approved by the Institutional Ethics Committees of the LMU Munich and the Medical University of Vienna.

### Laser‐capture microdissection

LCM experiments were performed as described before.^12^ We used FITC‐labeled anti‐perforin antibody (B‐D48; Abcam, Berlin, Germany),before a 1:20 diluted AF488‐labeled goat‐anti FITC antibody (AB_221558; Invitrogen, ThermoFisher, Darmstadt, Germany) was added together with anti‐CD8alpha antibody LT8 (1:20; Bio‐Rad, Munich, Germany) labeled using the Cy3 Labeling‐Kit (GE, Frankfurt, Germany). Sections were examined under 2‐propanol to identify CD8^+^ T cells with vectorial expression of perforin. Selection criterion was that two or more perforin granules were identified close to the cell membrane within an angle of 45° as calculated from the center of the cell. Of such CD8^+^ cells, the target area “T” opposing the perforin granules and the control area “C” on the opposite side of the T cell was isolated. Biological replicates were performed in two series of experiments (exp. 1 and 2). In both experiments, areas T and C of 51 CD8^+^ T cells with vectorial perforin orientation were pooled independently. Different compositions of biopsy tissue blocks from patient A were used: exp. 1: Block 10A back (45%), 11A front (45%), and 12B front (10%); exp. 2: Block 10A back (25%), 11B right (25%), 12A front (25%), and 12B back (25%). Percentages refer to the relative areas of the respective tissue block. In both experiments ~50 cm^2^ of tissue were used.

### RT‐PCR and NGS analysis

We amplified cell type‐specific transcripts by RT‐PCR and analyzed the amplicons by NGS.^24^ RNA was isolated from independently pooled isolated target and control areas using the PicoPure RNA isolation kit. Reverse transcription was performed, followed by two rounds of multiplexed first and second PCR of 25 cycles each using outer and nested inner primer pairs at 0.1 *μ*mol/L each. Primer sets were designed to amplify transcripts of the most common candidate cell types. Thus, mRNA specific to very rare cell types may not be detected. We used different primer sets in exp. 1 and exp. 2 which are listed in Table S1. Efficiencies of all HPLC purified primers were validated using RNA from human tonsils and brain. The amplicons were separated on 2% preparative agarose gel electrophoresis, bands with 250 ± 20 bp were isolated. A total of 50–75 ng were used for NGS library preparation with the Nugen Ovation Ultralow system for low comlexity samples. Libraries were sequenced at IMGM (Martinsried, Germany) by paired‐end 2 × 250 bp Illumina MiSeq sequencing. Raw sequencing data were demultiplexed based on the library‐specific barcodes and converted to FASTQ files with sequencing reads and quality scores using Illumina standard software (Illumina, Munich, Germany). Reads were then aligned to our customized database with the type‐specific target genes used in the RT‐PCR. Then, the number of reads matching each of the type‐specific transcripts was scored.

### Fluorescence IHC on frozen tissue sections

Ten micrometer thick tissue sections were air dried, fixed in acetone, washed in PBS, blocked in 5% BSA, and in biotin blocking buffer (Abcam) if biotin‐streptavidin amplification was used later. All antibodies were diluted in 1% BSA in Dako Wash Buffer (Dako, Hamburg, Germany), aside from anti‐CD11b which was diluted in 10% human serum (Sigma, Deisenhofen, Germany) and 2% BSA. After each incubation, the sections were washed in PBS.

The following primary antibodies were used in this study: mouse anti‐CD163 (EDHu‐1, dilution 1:200; Bio‐Rad), mouse anti‐CD8*α* (4B11, diluted 1:40; Bio‐Rad), rabbit anti‐CD11b antibody (EP1345Y, dilution 1:100; Abcam), and mouse anti‐perforin (B‐D48, dilution 1:100; Abcam). Anti‐CD163 was directly labeled using the Zenon AlexaFluor 647 mouse IgG1 labeling kit (Invitrogen). Binding of primary antibodies was detected using the following secondary antibodies (all Invitrogen): goat anti‐mouse IgG1 conjugated with AF488, goat anti‐mouse IgG2b conjugated with AF594, and goat antirabbit IgG conjugated with AF647. A biotin‐labeled secondary goat anti‐mouse IgG1 antibody and AF488‐labeled streptavidin were used for the detection of perforin. Nuclear staining was performed using DAPI (Sigma) and slides were mounted in Fluorescent Mounting Medium (Dako). Stainings were analyzed under a Axiovert 200 microscope (Zeiss, Munich, Germany). Each staining protocol was validated using appropriate isotype control antibodies.

### IHC on formaline fixed paraffin embedded (FFPE) tissue sections

For neuropathological classification, sections were stained with hematoxylin and eosin, luxolfast blue staining for myelin and Bielschowsky silver impregnation for axons. Imunocytochemistry was performed with a described biotin avidin technique.^9^, ^25^ Primary antibodies against the following targets were used: CD3, clone SP7 (Neomarkers, Fremont, CA, USA), Iba‐1 (Wako, Osaka, Japan), CD163, clone 10D6 (Novocastra, Wetzlar, Germany), myelin oligodendrocyte glycoprotein,^26^ myelin associated glycoprotein (AB B11F7),^27^ neurofilament (Chemicon, Darmstadt, Germany), and glial fibrillary acidic protein (Dako).

### Fluorescecnce IHC on FFPE tissue sections

Sections were deparafinized in Xylene and Ethanol and rinsed in water. Heat‐induced epitope retrieval with Tris‐EDTA, pH = 9 was performed for 2.5 h in a steamer. Sections were washed in TBS, permeabilized with 0.2% Tween, washed, and blocked for 1 h in 5% BSA (Jackson Immunoresearch, Hamburg, Germany) in TBS. An endogenous avidin/biotin blocking kit (Abcam) was used if biotin‐streptavidin amplification was employed. The same primary antibodies were used for FFPE as for frozen sections, aside from the mouse anti‐perforin antibody (5B10, dilution, 1:30; Dianova, Hamburg, Germany) and the rabbit anti‐CD163 antibody (EPR19518, dilution 1:200; Abcam). Isotype control stainings were performed to ensure specificities.

### Quantification of IHC

Images of MS lesions were quantified using ImageJ‐Fiji (https://imagej.net/Welcome). CD8^+^ T cells, CD163^+^ cells, and CD11b^+^ cells were counted in parenchymal and perivascular areas. CD163^+^ and CD11b^+^ double‐positive cells were only counted in areas where CD8^+^ T cells were visible. Ten to 20 fields of view were counted at 40×, depending on the density of the CD8^+^ T cells within the lesion, thereby covering an area of 1–50 mm^2^. Unless otherwise stated, median values of counts per mm^2^ are given.

## Results

**Figure 2:**
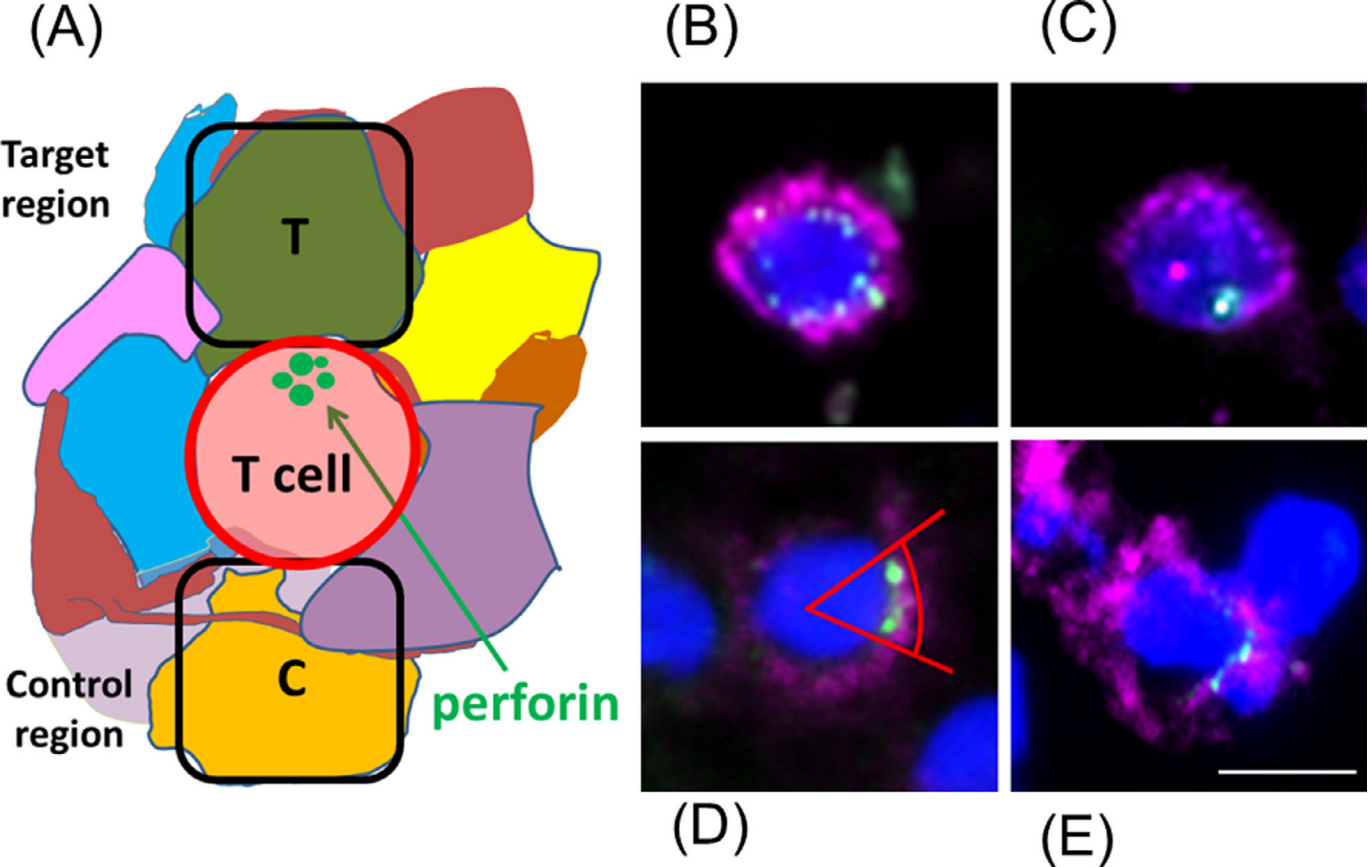
Strategy to identify the target cells of cytotoxic T cells in densely packed tissues. (A) Scheme of the experimental approach. A CD8^+^ T cell (red) is surrounded by many different cells of various type (different colors). Some cells may have long protrusions. We assume that a cytotoxic CD8^+^ T cell communicates with its target cell by orienting cytotoxic granules (green) toward the target cell. The area where a putative target cell is presumably located is indicated (area T). The control area was chosen at the opposite side of the T cell and is designated area (C). (B–D) Examples of different patterns of perforin‐containing granules. Tissue slices from an MS lesion of patient A were stained for CD8 (magenta), perforin (green), and nuclei (dark blue). Different patterns can be distinguished: (B) several perforin‐containing granules are distributed isotropically around the nucleus; (C) only one granule is seen; (D) several granules are accumulated at one side of the CD8^+^ cell an angle of 45° is indicated, which we chose as criterion for vectorial orientation; (E) same as (D), but with a flattened surface between T and target cell. This is regarded the strongest indication for specific interaction by an immunological synapse. Scale bar 10 *μ*m.

Our strategy was based on the assumption that a cytotoxic CD8^+^ T cell is surrounded by many cells of different type and that the vectorial orientation of cytotoxic granules in the T cell will indicate the direction of its cellular communication partner (see Fig. 2A). We isolated two regions by LCM: target‐region “T”, where the target cell is suspected, and control‐region “C” on the opposite side of the T cell. From both regions, we analyzed cell‐type‐specific transcripts by RT‐PCR and NGS. If a T cell, as hypothesized, communicate preferentially with a particular target cell type, specific transcripts of this cell‐type are expected to be enriched in region T as compared to region C. If vectorial orientation occurred just by chance, equal transcript levels would be detected in both regions T and C. Equal transcript levels would also be expected if the T cells interacted with thin protrusions of surrounding cells such as neurons, oligodendrocytes, or astrocytes, because such protrusions are present ubiquitously on all sides of the T cells. However, mRNA levels in protrusions, particularly in axons, may be low as compared to cellular somata and therefore escape detection.

We examined a frozen biopsy sample of index patient A, who underwent resection of a white matter lesion.^10^ The resectate underwent detailed neuropathological analysis (see “Patients and Methods” section, Fig. 1, and Table 1). It was shown previously^13^ that in this lesion CD8^+^ T cells outnumbered CD4^+^ cells by a factor of 8 in the parenchyma and that the overwhelming majority of the CD8^+^ cells are T cells because they also stained positive for CD3, which excludes macrophages, NK, or other CD8^+^ CD3^−^ cells. Most CD3^+^CD8^+^ T cells were of the memory‐effector CD45RO^+^ phenotype indicating that they had contacted antigens before. To detect vectorial perforin orientation in CD8^+^ T cells we triple‐stained sections from different regions of the section for CD8, perforin, and nuclei. When averaged over all regions of the sample, perforin was detectable in 44% of CD8^+^ cells with a detectable nucleus, but in the overwhelming majority of CD8^+^ T cells the perforin granules were distributed isotropically (Fig. 2B) or showed only one granule (Fig. 2C). We could identify few CD8^+^ cells that had two or more perforin‐positive granules oriented within an angle of 45° from the center of the T cell (Fig. 2D). Some of these cells showed flattened surfaces oriented toward their neighboring target cells (Fig. 2E).

We stained cryo‐sections from brain lesions of patient A for CD8 and perforin and isolated target and control areas of equal size by LCM. We exclusively collected areas which flanked CD8^+^ T cells that had two or more perforin granules located within an angle of 45° from the center of the T cell (Fig. 2D). T cells with vectorial perforin orientation were extremely rare and amounted to <3% of all CD8^+^CD3^+^ T cells, corresponding to about 1 cell per cm^2^ of the biopsy sample when averaged across all anatomical regions. We performed biological duplicates in two experiments (exp. 1 and exp. 2) by sampling from several tissue blocks with different amounts of active lesions (see “Patients and Methods” and Table 1). In each experiment we isolated 51 paired T and C regions, which we separately pooled and analyzed by RT‐PCR and NGS (see Table S1 for templates and primer pairs). We note that the only read‐numbers for identical template mRNAs are comparable among each other. Read‐numbers for different templates are not comparable because the initial mRNA levels (in terms of numbers of mRNA molecules per cell), the stabilities of different mRNA species, and the PCR amplification efficiencies may all vary over very broad ranges.

**Figure 3:**
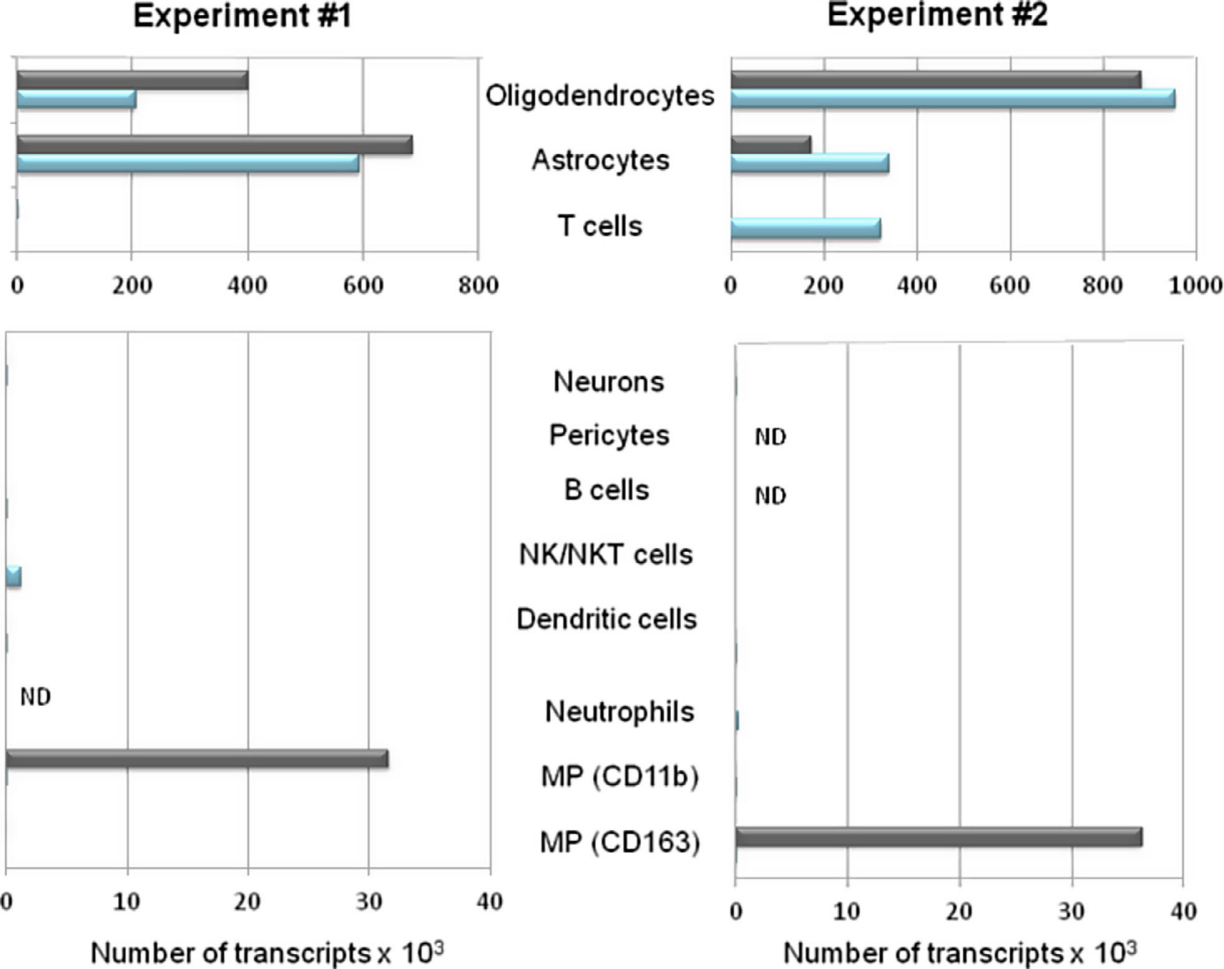
Signals of specific PCR products revealed by RT‐PCR and NGS. Numbers of transcripts of cell‐type specific PCR products for different cell types from biopsy sections of patient A are shown. Data for two independent experiments are shown. The absolute number of reads are comparable for identical template mRNAs but not for different templates. Slightly different primer sets and a different composition of anatomical locations were used in experiment 1 and 2. Transcript numbers of the target area T are shown in grey and from the control area C in blue. Signals from areas T and C were almost equal for oligodendrocytes‐ and astrocyte‐specific transcripts. In experiment 2, signals from T cells were observed in the control area C. Transcript numbers from all other cell types were very low or not detectable, except for MPs, where we found strong signals from CD11b in experiment 1 and CD163 in experiment 2. ND, not done.

For astrocytes and oligodendrocytes we observed reads from both regions T and C in both experiments (Fig. 3 upper panel) at about the same levels. These cell types are the most prevalent cells in the brain, have irregular shapes and many protrusions that vein the entire brain. Therefore it is expected that they are detected in all analyzed regions. No transcripts from T cells were observed in exp. 1 however in exp. 2 we found T cell transcripts in the control regions C (Fig. 3 upper panel). This is probably due to the fact that the tissue blocks used in exp. 2 contained higher proportions of lesions with tightly packed T‐cell infiltrates, thus increasing the probability of T‐cell detection. Furthermore, we note that isolation of cells by microdissection will never retrieve a single cell only. Instead it is expected that small shreds of other cells will be isolated together with larger volumes of the target cell (see Fig. 2A). Therefore it is almost certain that T‐cell transcripts will also be present in the target although their concentration was probably too low to be amplified to a level above our detection threshold.

From many candidate cell types, such as neurons, pericytes, granulocytes, B, NK, and NKT cells we observed no or just marginal signals (Fig. 3 lower panel). Of note, only for the two transcripts CD11b in exp. 1 and CD163 in exp. 2, we observed significantly higher signals in region T as compared to region C. CD11b and CD163 are both markers for MPs, such as microglia, macrophages, and monocytes. CD11b (Integrin alpha‐M) belongs to the integrin‐alpha family, which is up‐regulated in chronic MS‐plaques,^28^ and has a broad expression spectrum including all MPs, granulocytes, and NK cells.^29^ CD163 is a scavenger receptor for hemoglobin‐haptoglobin complexes and is also expressed in M2 polarized macrophages in vitro.^25^ Four reasons may explain why we found CD11b in exp. 1 and CD163 in exp. 2: First, CD163 was only expressed in a subset of MPs and the numbers of CD163^+^ cells was highly variable between different tissue blocks and areas (Fig. 1J–L). Second, the distribution of CD11b+high and CD11b+weak cells varied significantly within the lesion. Third, the composition of PCR primers in exp. 2 differed slightly from exp. 1 (Table S1). After exp. 1 had revealed MPs as most likely targets of the CD8^+^ T cells, we deleted primers for B cells and pericytes from the multiplex PCR primer set of exp. 2 and included more primers for transcripts specific for MPs. Hence, the different composition of the multiplex PCR primer pool might have influenced PCR efficiencies. Fourth, although RT‐PCR and NGS are sensitive techniques, partial mRNA degradation in the archival tissue might have prevented that the detection threshold was reached for CD163 in exp. 1 and CD11b in exp. 2. However, both independent experiments suggested MPs as target cells of CD8^+^ T cells in an unbiased experimental setup.

**Figure 4:**
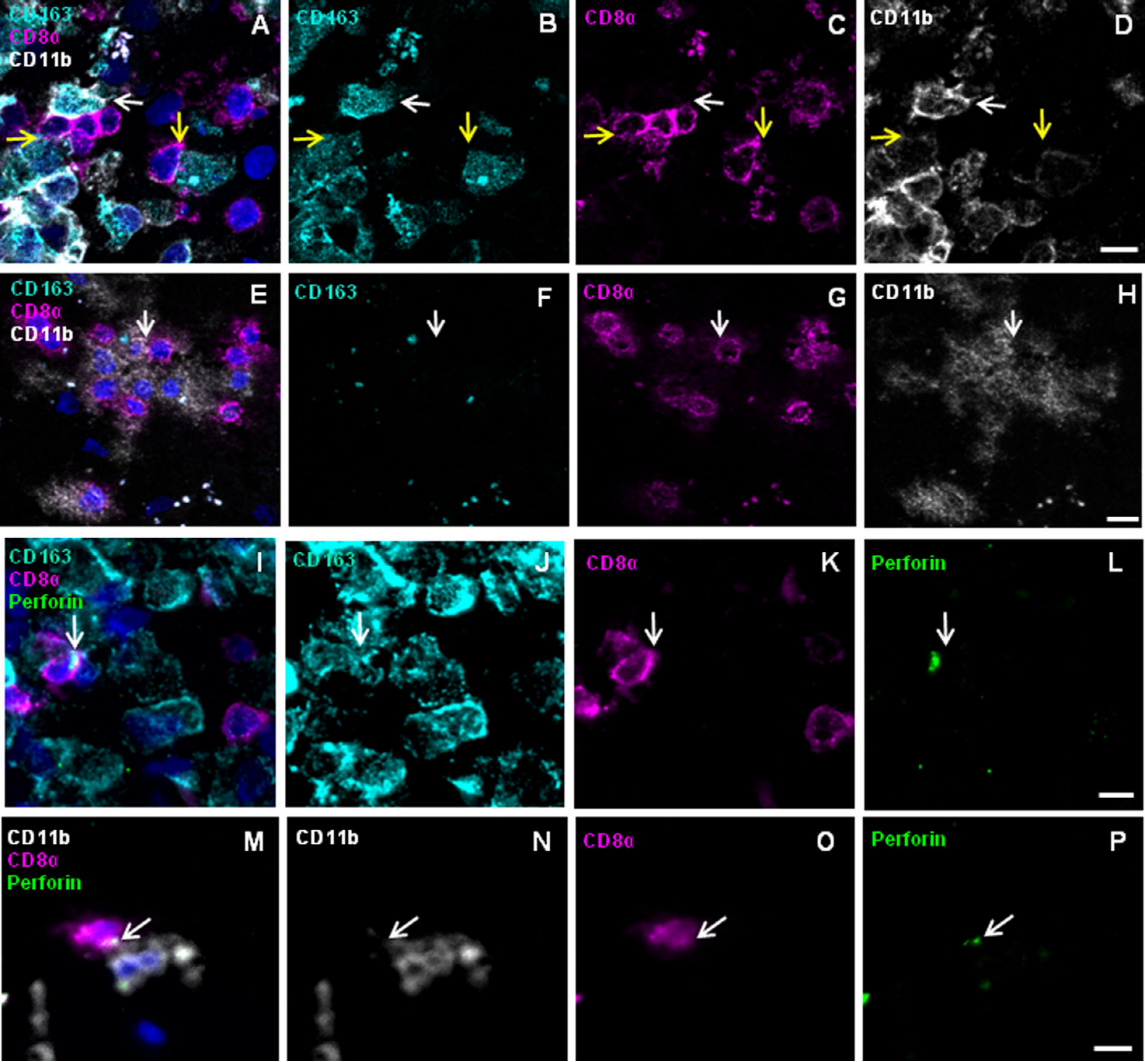
Immunohistochemistry reveals CD8^+^ T cells juxtaposed to CD163^+^ and CD11b^+^ MPs showing vectorial perforin orientation in patient A. Frozen sections of brain lesions of patient A were stained for the markers CD8 (magenta), CD163 (cyan), CD11b (white), perforin (green), and nuclei (DAPI, dark blue). Rows 1 and 2 show the four‐color overlay of CD163, CD11b and CD8 (A and E) and single color staining for each of the antigens CD163 (B and F), CD8 (C and G), and CD11b (D and H). CD8^+^ cells were detected in close physical contact with CD163^+^ CD11b^high+^ as well as with CD163^+^ CD11b^weak+^ cells (A–D) as indicated by white and yellow arrows, respectively. Furthermore, CD8^+^ cells contacted CD163^−^ CD11b^+^ cells (E–H). Rows 3 and 4 show the four‐color overlay of CD8 and perforin with either CD163 (I) or CD11b (M) and single color staining for each of the antigens CD163 (J), CD11b (N), CD8 (K and O), and perforin (L and P). Arrows indicate accumulations of perforin‐containing granules. (I–L) One of several CD8^+^ cells orients big perforin granules toward a CD163^+^ cell. The CD8^+^ cell has a round shape and the granules are in the cytosol between the nucleus and the cytoplasmic membrane. (M–P) CD8^+^ cell orients perforin granules toward a CD11b^+^ cell. Scale bars 10 *μ*m.

We confirmed perforin‐mediated communication between CD8^+^ cells and CD163^+^ or CD11b^+^ MP in acute lesions of patient A by 4‐color IHC (Fig. 4). CD8^+^ cells can be detected juxtaposed to CD163^+^CD11b^high+^ cells (Fig. 4A–D, white arrow) and to CD163^+^CD11b^weak+^ cells (yellow arrows). In some instances, we detected two or more CD8^+^ T cells interacting with CD163^+^CD11b+ cells (Fig. 4A–D, upper left region). Furthermore, a cluster of CD8^+^ cells in contact with CD163^−^CD11b^+^ cells was detected (Fig. 4E–H). Different CD11b staining intensities probably reflect expression of CD11b on a variety of cellular subtypes. Staining for CD8, CD163, perforin, and nuclei reveals a CD8^+^ T cell with perforin oriented vectorially toward a CD163^+^ target cell (Fig. 4I–L). Staining for CD8, CD11b, perforin, and nuclei shows a CD8^+^ T cell that orients perforin vectorially toward a CD11b^+^ cell (Fig. 4M–P). Although IHC may only spotlight few exemplary situations, these data illustrate that CD163 and CD11b expressing MP cells communicate with CD8^+^ T cells in an acute MS (AMS)‐lesion of patient A and thus confirm our LCM experiment.

**Figure 5:**
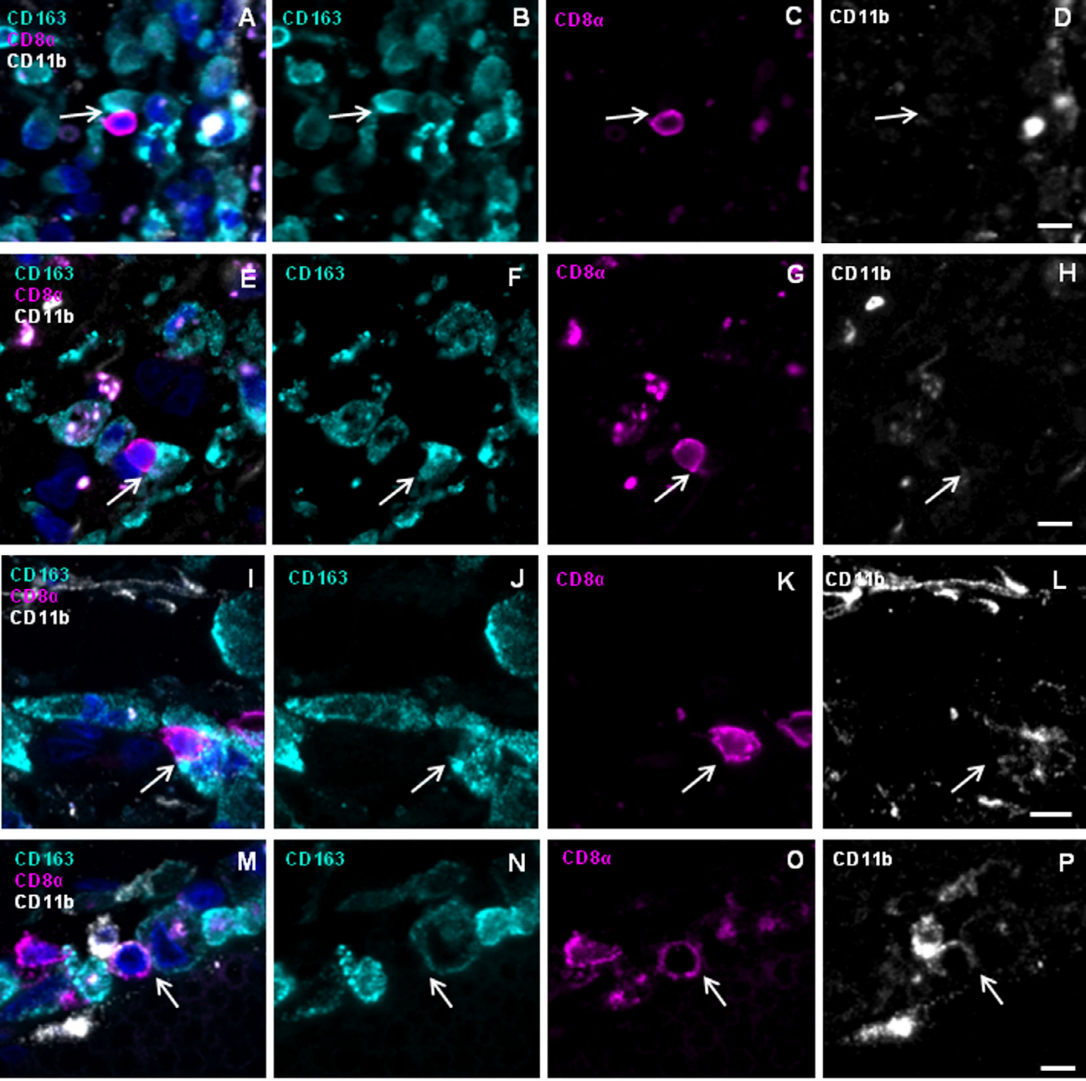
Immunohistochemistry reveals CD8^+^ T cells in direct contact with MPs in other MS patients. FFPE fixed tissue sections of brain lesions of patients MS11 perivascular (A–D), MS11 parenchymal (E–H), MS10 parenchymal (I–L), and MS25 perivascular (M–P) were stained for the markers CD163 (turquois), CD8 (magenta), CD11b (white), and nuclei (DAPI, dark blue). We show the four‐color overlay (A, E, I, and M) and single color staining for each of the antigens CD163 (B, F, J, and N), CD8 (C, G, K, and O) and CD11b (D, H, L, and P). Arrows indicate contact between a CD8^+^ T cell with flattened surface and CD163^+^ cell. Note the weak expression of CD11b on CD163^+^ cells (D, H, L, and P), but strong CD11b expression on CD163^−^ cells (L and P). Scale bars 10 *μ*m.

To investigate whether interactions of CD8^+^ T cells with MPs are peculiar to patient A or may be observed in other patients, we investigated a well characterized cohort of patients with MS and other inflammatory neurological diseases. Because further LCM experiments would require freshly frozen samples of sufficient size and mRNA preservation, which are un available, we used IHC to examine five further cases of AMS, one case with primary and two cases with secondary progressive MS (PPMS, SPMS), and one case each of Rasmussen encephalitis (RE), and neuromyelitis optica (NMO) (for patient details see Patients and Methods and Table 1, columns 1–3). In all patients we found perivascular and parenchymal, CD163^+^ and CD11b^+^ cells as well as infiltrates of CD8^+^ cells (Table 1, columns 4–9). The only exceptions were patients MS4 and RE1, where we did not find CD163 cells in the parenchyma. In AMS, patients A and MS11 showed the highest numbers of perforin expressing cells and the numbers of CD163^+^ cells correlated with the numbers of CD8^+^ cells. Overall, the distribution of cells between parenchyma and perivascular regions, varied considerably between different patients. In all patients, except patients MS4, MS1854, and RE1, we observed CD163^+^ and CD11b^+^ cells in direct contact to CD8^+^ cells by 4‐color staining for CD8, CD163, CD11b, and nuclei (Fig. 5, Table 1, column 10).

**Figure 6:**
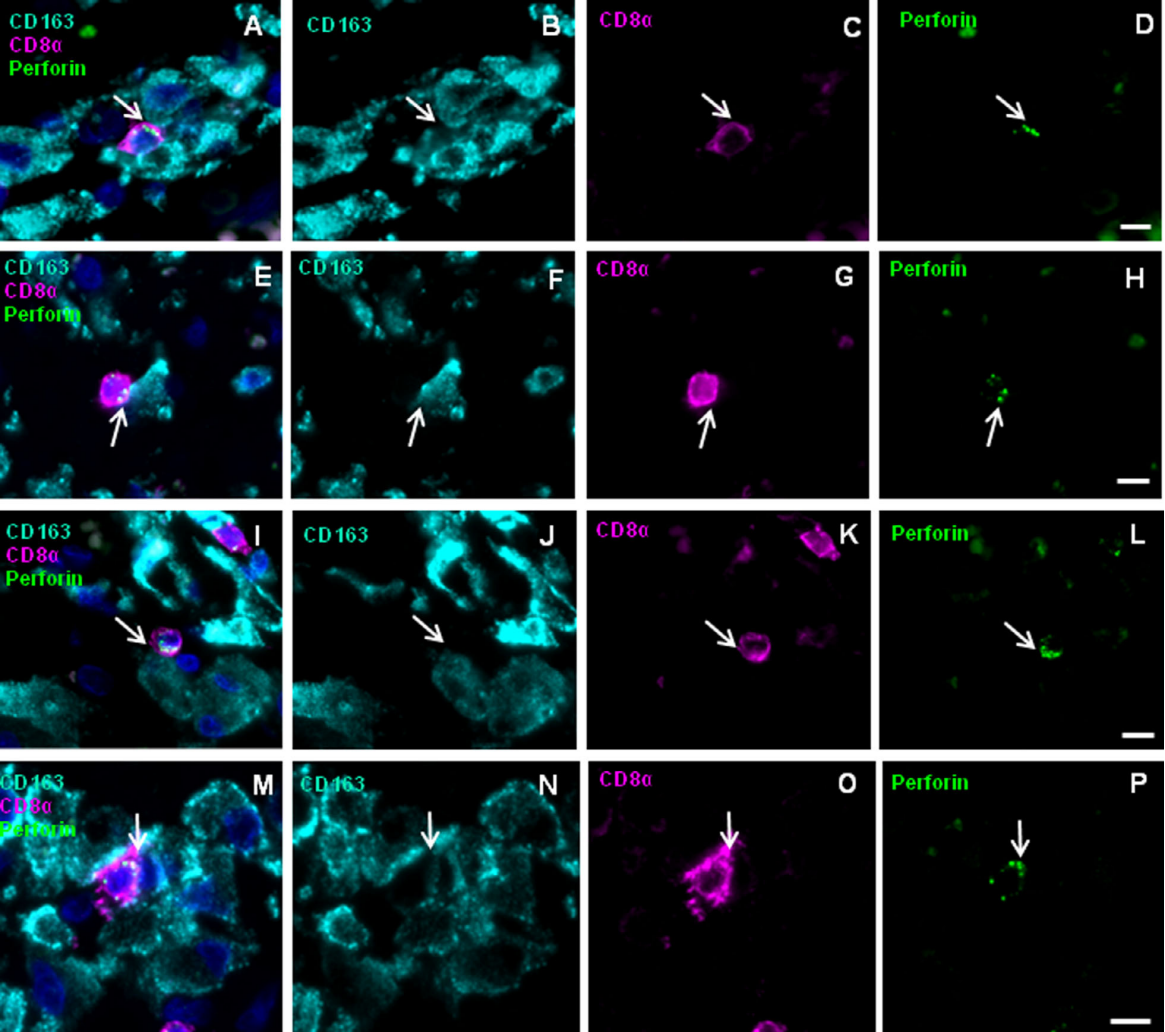
Vectorial perforin orientation of CD8^+^ cells toward CD163^+^ MPs in brain lesions of other patients with MS. FFPE fixed tissue sections were stained for the markers CD163 (turquois), CD8 (magenta), perforin (green), and nuclei (DAPI, dark blue). Shown are a perivascular region of AMS patient MS11 (A–D), a parenchymal region of MS11 (E–H), a perivascular region of AMS patient MS10 (I–L), and a perivascular region of SPMS patient MS25 (M–P) The four‐color overlay (A, E, I, and M) and single color staining for each of the antigens CD163 (B, F, J, and N), CD8 (C, G, K, and O) and perforin (D, H, L, and P) is shown. Arrows indicate accumulations of perforin‐containing granules toward CD163^+^ cell. Scale bars 10 *μ*m.

Whenever the numbers of CD163^+^ cells in the parenchyma of MS patients exceeded ~120 cells/mm^2^, we could detect CD163^+^ cells in direct contact with CD8^+^ cells that were positive for perforin (Table 1, columns 10 and 11). This was observed by four‐color staining for CD8, CD163, perforin, and nuclei for two blocks of patient A (Fig. 4), the two AMS cases MS11 (Fig. 6A–H) and MS10 (Fig. 6I–L), and case MS25 with SPMS (Fig. 6M–P). Contacts were detected in a perivascular (A–D) and a parenchymal region (E–H) of AMS patient MS11, a perivascular region of AMS patient MS10 (I–L), and a perivascular region of SPMS patient MS25 (M–P). Possibly such contacts might also be observed in other patients, but the low frequencies of CD8^+^ and CD163^+^ cells may have precluded detection. Of note, we did not detect pairs of CD163^+^ cells in contact with perforin‐expressing CD8^+^ cells in the NMO case, despite the high number of parenchymal CD163^+^ cells. In several patients we observed vectorial perforin orientation of CD8^+^ T cells toward CD163^+^ target cells. These examples show that communication of CD8^+^ T cells with CD163^+^ MPs by vectorial perforin orientation is not exclusively detectable in patient A, but may also be observed in other patients with AMS and SPMS.

## Discussion

To identify the communication partners of CD8^+^ T cells in MS brain lesions, we developed an unbiased strategy that uses vectorial perforin orientation in T cells as guidepost for localizing their target cells without the need to visualize the targets directly. By excising areas juxtaposed to vectorially oriented perforin granules and testing transcript signatures of many candidate cell types, we could identify MPs as the target cells of cytotoxic CD8^+^ T cells in an index MS patient. Using IHC, we then validated this result and confirmed it in other patients with active MS lesions in situ. This approach may be applicable to any tissue even if it is tightly packed with various cell types.

Analysis of specific interactions of T cells with their target cells in tightly packed tissue by classical IHC is limited to unusual conditions where the targets are known a priori, for example in tumors, where only few candidate targets are present, for example in muscle fibers,^19^ or where dense cell packing is loosened, for example in edema.^20^, ^30^ However, in many disorders, in particular in autoimmune diseases, the target cells of cytotoxic T cells remain enigmatic. Our strategy overcomes this limitation because we isolate the putative target cells “blindly” and identify the target cells by RT‐PCR and NGS. To this end, we used PCR primers specific for bona fide cell‐type specific transcripts but in case very well preserved tissue is available, whole transcriptome analyses may be performed. Unfortunately, RNA‐preservation of our brain specimens did not allow this, due to partial RNA degradation. Some primer pairs did not yield detectable amplicons because the detection threshold was not overcome. This may have had several reasons: First, we used archival tissue with a priori limited RNA preservation. Second, we could not fix the tissue by aldehydes because this would have hampered PCR. Third, the tissue had to be stained by fluorescent antibodies prior to microdissection, which gives rise to further RNA degradation by RNase. Fourth, we were restricted by very low cell numbers because CD8^+^ T cells with vectorial perforin orientation were very scarce. Therefore, large areas had examined, which lead to additional RNA degradation. Fifth, many candidate transcripts that were bona fide cell type specific are expressed at low rates. Sixth, to amplify several candidate transcripts in one reaction, we had to use multiplex PCR, where it is known that some primers may hamper amplification of other templates. Therefore we have confirmed interaction of CD8^+^ T cells with CD11^+^ and CD163^+^ MPs by classical IHC, a technique that is independent of the intricacies of molecular methods. This interaction was not only seen in classical active lesions with very dense macrophage infiltration, but also in initial “prephagocytic” lesions with some microglia activation, but very low density of infiltrating macrophages.

Very low concentrations of template mRNA together with shortcomings of multiplex PCR, in particular with different primer sets in exp. 1 and 2, were probably also the reason why we identified CD11b^+^ cells in exp. 1 and CD163^+^ cells in exp. 2 as communication partners of perforin excreting T cells. Furthermore, in exp. 1 and 2, partly different tissue blocks were used and the inflammatory status within a block changed rapidly within a few micrometers. Therefore the distribution of CD11b^+^ and in particular of CD163^+^ cells was highly inhomogeneous in all blocks. This is expected as gene expression varies considerably between acute and chronic lesions.^28^ However, both, CD11b and CD163, are characteristic markers for MPs. This suggests strongly that MPs are target cells of the perforin excreting CD8^+^ T cells in MS lesions of patient A, as we found strong signals from these transcripts in the target areas, but not in control areas in two independent experiments. By contrast, we did not detect signals from dendritic cells, neurons, pericytes, granulocytes, B, NK, and NKT cells. Oligodendrocytes and astrocytes are also unlikely targets because we observed strong signals in both, the target and in the control area, probably because both cell‐types belong to the most frequent cells in brain white matter tissue and are of irregular shape so that mRNA may be present virtually everywhere, even in a tiny segment of a microdissected sample. Furthermore, both cell types have many long and thin protrusions which make physical contacts of T cells with oligodendrocyte‐ and astrocyte‐protrusions unavoidable. Therefore, we cannot completely exclude that also some functional interactions with T cells may take place.^30^ However, such interactions could not be detected here, in contrast to interactions between T cells and MPs.

MPs are a highly plastic population of different, though closely related cell types, which often convert into each other depending on the local milieu and activation status in particular in MS.^15^, ^16^ Currently we cannot yet pinpoint the precise subtype of MPs that interacts with CD8^+^ T cells in the MS lesions; however, we assume that CD8^+^ T cell may communicate with several subtypes because we found them in contact with CD163^+^CD11b^+^ and CD163^−^CD11b^+^ cells. CD11b is a classical marker for all microglia and macrophage subtypes which is expressed in all regions of human brain.^31^ CD163 is usually found on perivascular macrophages in the normal brain^25^, ^32^, ^33^ but also on parenchymal microglia and macrophages in Alzheimer's and Parkinson's diseases^34^ and MS.^25^, ^32^ Here we found variable numbers of CD163^+^ and CD11b^+^ cells at perivascular sites and in the parenchyma of all but two patients (Table 1). This agrees with an earlier study, which located CD163^+^ cells at highly variable levels in initial, early, and late active lesions of MS brain.^25^ As CD163 is a marker for M2 macrophages, which are involved in tissue repair and have antiinflammatory function, the interaction of CD163^+^ MPs with T cells might possibly calm inflammation. However, CD163 is also a scavenger receptor for the hemoglobin‐haptoglobin complex.^35^ This is of particular interest because iron metabolism is known to be disturbed in MS lesions^36^ and highly reactive oxygen radicals catalyzed by free iron might create neo‐antigens by chemical modification of peptides. This, by contrast, might have proinflammatory function. Thus, CD163 obviously plays an important role in MS pathogenesis, but its precise function remains so far enigmatic.

It is clear that our observations in human tissue cannot provide formal proof that CD8^+^ T cells communicate exclusively with myeloid cells, in particular as to date the target antigens of the invasive T cells are unknown. We cannot rule out that MPs incidentally happen to be close to the immunological synapse of CD8 T cells, maybe for clearing debris from recently destroyed cells. Lesional MPs may contain myelin fragments,^23^ but it is not known whether such myelin antigens are indeed recognized. In an animal model,^37^ T cell reactivity toward MPs could be enhanced by anti‐myelin antibodies that were bound by MPs, but comparable evidence is missing in humans. Furthermore, vectorial perforin orientation does not necessarily implicate killing of the target cell. Halle et al.^38^ showed that in vivo the “killing efficiency” was orders of magnitude less than in vitro. This means that most of the specific T cell‐MP contacts will probably not result in the immediate destruction of the target cells. Regardless of whether the granules are secreted or retained in the cytoplasm, their vectorial orientation strongly suggests that the T cell has formed an immunological synapse with the neighboring MP. The functional consequences of this interaction could be manifold. The T cell may be activated and presumably proliferate, which is consistent with previously observed clonal expansions of CD8^+^ T cells.^8^, ^11^, ^12^ The activated T cells might act as effectors and kill certain target cells or produce proinflammatory cytokines and mediators, thereby inciting the inflammatory milieu. Alternatively, they might dampen local inflammation. These processes might not be mutually exclusive but might change their character with the location within the lesion and during the course of disease. Our observations may pave the way for new therapeutic approaches designed specifically to target local CD8^+^ T cell‐MP interactions.

In conclusion, by applying a novel approach to discover the cellular interaction partners of brain‐infiltrating CD8^+^ T cells in MS brain lesions, we found that CD8^+^ T cells interact with CD11b or CD163‐expressing MPs. Although the patient cohort is not very large and we could not detect vectorially perforin excreting CD8^+^ T cells in all patients, our finding in this pilot experiment underscores the pathogenic importance of CD8^+^ T cells in MS. Our methodological approach should be applicable to pathological specimens from a wide range of human conditions.

## Author Contributions

M.K.S. performed most LCM, PCR, and IHC experiments. K.H. performed LCM, PCR, and IHC experiments. E.B. performed PCR and NGS experiments. A.N. performed analysis of the biopsy specimens from patient A. E.M. provided samples and clinical data. R.H. partly designed the research and contributed to data analysis and writing. H.L. provided samples, analyzed, and characterized human biopsy and autopsy samples, and wrote part of the manuscript. K.D., M.K.S., K.H., E.B., and H.L. analyzed the data. K.D. initiated and designed the research and wrote the paper. All authors edited and reviewed the manuscript.

## Conflict of Interest

None declared.

## Supporting information


**Table S1.** List of transcripts employed for target cell analysis and of primers used for amplification of transcripts by RT‐PCR.
