# Supplementary material for: Communication of CD8+ T cells with mononuclear phagocytes in multiple sclerosis
Source: Ann Clin Transl Neurol. 2019 Jun 14;6(7):1151–64. doi: 10.1002/acn3.783 (PMC6649540; doi:10.1002/acn3.783)
Supplement: Supplementary file 1 — Table S1. List of transcripts employed for target cell analysis and of primers used for amplification of transcripts by RT‐PCR. [file ACN3-6-1151-s001.pdf]

Supplementary Materials

Communication of CD8$^+$ T cells with mononuclear phagocytes in multiple sclerosis brain lesions

Short title: Target cells of CD8$^+$ T cells in multiple sclerosis

Matea Konjevic Sabolek, Kathrin Held, Eduardo Beltrán, Anna G. Niedl, Edgar Meinl,

Reinhard Hohlfeld, Hans Lassmann, and Klaus Dornmair

Supplemantary Table S1. List of transcripts employed for target cell analysis and of primers used for amplification of transcripts by RT-PCR.

Transcripts tested in both experiments are unmarked, transcripts tested only in exp#1 are indicated with one asterisk (*), and transcripts tested only in exp#2, with two asterisks (**).

Primers designated with "For"(ward) elongate the coding strand and primers designated with "Rev"(erse) elongate the non-coding strand of DNA. All primers were designed to give final product of 240 to 260bp in size. The appendix "out" refers to the outer primers in nested PCRs. The appendix "in" refers to the inner primers in nested PCRs. All inner primer pairs were designed to give final products of 240 to 260 bp.

Transcripts

Cell-type	Transcript	UniProt ID
Neurons	GABRA1	P14867
Neurons	SNAP25	P60880
Neurons	STMN2	Q93045
Astrocytes	GFAP	P14136
Astrocytes	AQP4	P55087
Astrocytes	SLC1A2	P43004
Oligodendrocytes	MOG	Q16653
Oligodendrocytes	MAG	P20916
Oligodendrocytes	UGT8	Q16880
Microglia/macrophages	P2RY12	Q9H244
Microglia/macrophages	HEXB	P07686
Microglia/macrophages, DC	C1QA	P02745
Microglia/macrophages, neutrophil, NK cells	CD11b (ITGAM)	P11215
Microglia/macrophages	CD68	P34810
Macrophages	CD163	Q86VB7
DC	LILRA4 (ILT7)*	P59901
DC, macrophages	ZNF366	Q8N895
DC	CD1A*	P06126
Endothelial cells	DES*	P17661
Endothelial cells	MCAM (CD146)*	P43121
Endothelial cells	CSPG4*	Q6UVK1
T cells	CD3D	P04234
T cells	CD28	P10747
T cells, macrophages	CD4	P01730
B cells	CD19*	P15391
B cells	MS4A1 (CD20)*	P11836
B cells	CD22*	P20273
NK cells, T cells	KLRB1 (CD161)*	Q12918
NK cells	CD244	Q9BZW8
NK cells	NCR1 (CD335)	O76036
DC	CD1C**	P29017
DC	CLEC4C (CD303)**	Q8WTT0
NK cells, macrophages, neutrophils	FCGR3A (CD16)**	P08637
Macrophages/microglia	FCGR1A (CD64)**	P12314
Macrophages/microglia	CD14**	P08571
Macrophages/microglia	MRC1 (CD206)**	P22897
Macrophages/microglia	MSR1 (CD204)**	P21757
DC	CLEC4A (CD367)**	Q9UMR7
Neutrophils,eosinophils	CEACAM8 (CD66B)**	P31997
Eosinophils	AZU1**	P20160
Eosinophils	ELANE**	P08246
NK cells	KLRF1**	Q9NZS2

Primers used for amplification of transcripts by RT-PCR.

Name	Sequence
GABRA1 - For out	5´ -GAG TAA CAA CTG TGC TCA CC- 3´
GABRA1 - Rev out	5´ -GAC AGT CGG TCA ATT TTG CTG- 3´
SNAP25 - For out	5´ -GAC GGA CCT AGG AAA ATT CTG- 3´
SNAP25 - Rev out	5´ -CTG TCG ATC TGG CGA TTC TG- 3´
STMN2 - For out	5´ -GAA GCC CCA CGA ACT TTA GC- 3´
STMN2 - Rev out	5´ -GAC CAG CTT TTC CTG CAG AC- 3´
GFAP - For out	5´ -TAT GAG GCA ATG GCG TCC AG- 3´
GFAP - Rev out	5´ -ATC TCG TCC TTG AGG CTC TG- 3´
AQP4 - For out	5´ -GAT GTC ACT GGC TCA ATA GC- 3´
AQP4 - Rev out	5´ -TCA ATC ACA TGC ACC ACT CC- 3´
SLC1A2 - For out	5´ -CAG GTG GAA GTG CGA ATG C- 3´
SLC1A2 - Rev out	5´ -CTG CAG CAA TGA TGG TCG TG- 3´
MOG - For out	5´ -GGG AAA GGT GAC TCT CAG G- 3´
MOG - Rev out	5´ -GAT GAT CAA GGC AAC CAA GG- 3´
MAG -For out	5´ -CAG TGA ACG GGA CAA TGG TG- 3´
MAG - Rev out	5´ -CTC GTT CAC GGT CAC ATT GC- 3´
UGT8 - For out	5´ -AAC TGG CCT TTG GTA TCC TG- 3´
UGT8- Rev out	5´ -GCT GGC TGG TTT GGT TAG G- 3´
P2RY12 - For out	5´ -CAA ACC CTC CAG AAT CAA CAG- 3´
P2RY12 - Rev out	5´ -CAA AAG TTC TCA GTG GTC CTG- 3´
HEXB - For out	5´ -GTC AGA GTG TGA TGC TTT CC- 3´
HEXB - Rev out	5´ -CTC AGG AAA AGT GAT GCT CTG- 3´
C1QA - For out/in	5´ -TAT CGC TGG CCT CTA TGG TG- 3´
C1QA - Rev out	5´ -GTC GAA GAT GAC CAC GTT GC- 3´
CD11b - For out	5´ -GCC AGT ACT GAG AGT CAA GG- 3´
CD11b - Rev out	5´ -TGG GTC CTC GAT GCA ATT CG- 3´
CD68 - For out	5´ -GGG CAT CTC TGT ACT GAA CC- 3´
CD68 - Rev out	5´ -TGG GGC AGG AGA AAC TTT GC- 3´
CD163 - For out	5´ -TTC CTT GTG GGA TTG TCC TG- 3´
CD163 - Rev out	5´ -CTG AAA TCA GCT GAC TCA TGG- 3´
LILRA4 - For out	5´ -CAT AAC CCC GTG ACC ATC TG- 3´
LILRA4 - Rev out	5´ -AGA GTG AAC CTG CCC AGT C- 3´
ZNF366 - For out	5´ -CGC GAG AAC ATC TGT GTG G- 3´
ZNF366 - Rev out	5´ -CAC GTG TCG CTT CAT GTT GG- 3´
CD1A - For out	5´ -CTT GTG TGC CAT GTC TCA GG- 3´
CD1A - Rev out	5´ -AGC GTT TCC TGA ACC AAA GC- 3´
DES - For out	5´ -AGA TGC GGG AAT TGG AGG AC- 3´
DES - Rev out	5´ -ATC CCG TGT CTC GAT GGT C- 3´
MCAM - For out	5´ -TGG AGA CAG GTG TTG AAT GC- 3´
MCAM - Rev out	5´ -TCT TCT GGG AGC TTA TCT GAC- 3´
CSPG4 - For out	5´ -CGG ACA CTT CTT CCG AGT G- 3´
CSPG4- Rev out	5´ -GAC AGG CAG CCT CAA AAG AC- 3´

CD3D - For out	5´ -CCT TCT CTC GCA AGT GAG C- 3´
CD3D - Rev out	5´ -CAG CCT TCC AGT CTC ATG TC- 3´
CD28 - For out	5´ -AGT CGC CCA TGC TTG TAG C- 3´
CD28 - Rev out	5´ -GGT GTT TCC CTT TCA CAT GG- 3´
CD4 - For out	5´ -TTA GGC ACT TGC TTC TGG TG- 3´
CD4 - Rev out	5´ -TTG GCA GTC AAT CCG AAC AC- 3´
CD19 - For out	5´ -TTC CAC CTG GAG ATC ACT GC- 3´
CD19 - Rev out	5´ -GCT CGG GTT TCC ATA AGA CG- 3´
MS4A1 - For out	5´ -GAA AGG CCC TAT TGC TAT GC- 3´
MS4A1 - Rev out	5´ -CCA GAA ATG GCA GCA AAG AG- 3´
CD22 - For out	5´ -CAG CTG AAC GTG AAG CAC AC- 3´
CD22 - Rev out	5´ -ACT TGA CTT CCC TCC ACA GC- 3´
KLRB1 - For out	5´ -GAT GTC TGT CAG GGT TCA CC- 3´
KLRB1 - Rev out	5´ -TGT CAC GTA TCA GGT TCT GTG- 3´
CD244 - For out	5´ -ATC CAG ACA GCA GGG AAC C- 3´
CD244 - Rev out	5´ -AAA GTC TGC TCC TGC TCG TG- 3´
NCR1 - For out	5´ -CAG CAG ACT CTC CCA AAA CC- 3´
NCR1 - Rev out	5´ -GAG ATC ACT TCG GGT CCA G- 3´
CD1C - For out	5´ -TGA AGG CGT CAC AGA AAC AG- 3´
CD1C - Rev out	5´ -ATC ACC AAG GGC ACT ATC AC- 3´
CLEC4C - For out	5´ -GCG TCA TGG AAG GAA AGG AC- 3´
CLEC4C - Rev out	5´ -ATT CCA GCC CCA TTC TTC TG- 3´
FCGR3A - For out	5´ -CAT ATC GGC TGG CTG TTG C- 3´
FCGR3A - Rev out	5´ -CCA AGC AGA AAG AGA CTT GG- 3´
FCGR1A - For out	5´ -GCT ATT TCC AGC TCC AGT GC- 3´
FCGR1A - Rev out	5´ -CCA CTG CCA GAT AGA AAA GG- 3´
CD14 - For out	5´ -TGC CAA CAG ATG AGG TTC AC- 3´
CD14 - Rev out	5´ -GAG GTT CGG AGA AGT TGC AG- 3´
MRC1 - For out	5´ -AGC GGA GAC ATA CTG CAA GC- 3´
MRC1 - Rev out	5´ -ATG CTG TGT GAT CTG ACT CC- 3´
MSR1 - For out	5´ -GTC CTC CGG GTC TTA AAG G- 3´
MSR1 - Rev out	5´ -CCA AAG TGA GCT GCC TTG TG- 3´
CLEC4A - For out	5´ -TAT GCC CGT GGA AGA GAC AG- 3´
CLEC4A - Rev out	5´ -TGA CCT TTG AGG ACC AAG AC- 3´
CEACAM8 - For out	5´ -CCA GCG AGT GCA AAC TTC AG- 3´
CEACAM8 - Rev out	5´ -ATG CTG ACA GTG GCT CTA GC- 3´
AZU1 - For out	5´ -CCC CTT TTG GAC ATC GTT GG- 3´
AZU1 - Rev out	5´ -AGG CAG TGG CAG TAT CGT C- 3´
ELANE - For out	5´ -CGT GAT TCT CCA GCT CAA CG- 3´
ELANE - Rev out	5´ -GGA TGA TAG AGT CGA TCC AG- 3´
KLRF1 - For out	5´ -AAG GAC CTT TGT GCT TCG AG- 3´
KLRF1 - Rev out	5´ -CAC TGC TGC AGG TTT CAG AG- 3´

Primers used for amplification of transcripts by nested PCR.

Name	Sequence
GABRA1 - For in	5´ -TGC CTT TGT GTT CTC AGC TC- 3´
GABRA1 - Rev in	5´ -TCG GGC TTG ACC TCT TTA GG- 3´
SNAP25 - For in	5´ -TGT GTG TCC CTG TAA CAA GC- 3´
SNAP25 - Rev in	5´ -CGA TCT CAT TGC CCA TAT CC- 3´
STMN2 - For in	5´ -CCA AAG AAG AAA GAC CTG TCC- 3´
STMN2 - Rev in	5´ -TAG CAG CTA GAT TAG CCT CAC- 3´
GFAP - For in	5´ -ACA TGC ATG AAG CCG AAG AG- 3´
GFAP - Rev in	5´ -CAG CGC CTC CTG ATA ACT G- 3´
AQP4 - For in	5´ -CTG GTG CCA GCA TGA ATC C- 3´
AQP4 - Rev in	5´ -AGA ATC AGG TCA TCC GTC TC- 3´
SLC1A2 - For in	5´ -CGA CAG TCA TCT TGG CTC AG- 3´
SLC1A2 - Rev in	5´ -ACC CTG TGA TTA AGC TGG AG- 3´
MOG - For in	5´ -CAA GAG GAG GCA GCA ATG G- 3´
MOG - Rev in	5´ -CCG GCA CAA TTA CAA ACA GG- 3´
MAG - Rev in	5´ -CTC CAC AGA CAG GTT GAA GG- 3´
UGT8 - For in	5´ -TCC TGC TCC ATT AGC ATA CG- 3´
UGT8 - Rev in	5´ -GGG AAT TCC AGT GCT ACG TC- 3´
P2RY12 - For in	5´ -GTC GAC AAC CTC ACC TCT G- 3´
P2RY12 - Rev in	5´ -TGT TCC CAG TTT GGC ATC AC- 3´
HEXB- For in	5´ -CTT AAG GCC AAC AGA GTT TGG- 3´
HEXB - Rev in	5´ -GAC TGG TCA TCA ACT ATG TGC- 3´
C1QA - Rev in	5´ -GCC TTT AAT TCC CGG GAT GC- 3´
CD11b - For in	5´ -GAG TGT AAT GAT CAG GTG GTG- 3´
CD11b - Rev in	5´ -CAA CTG TAG TTT CAG GGT CTC- 3´
CD68 - For in	5´ -AAG GTC CAG GGA AGC TGT G- 3´
CD68 - Rev in	5´ -GGT GAA AGA ATG ATG CTC GAG- 3´
CD163 - For in	5´ -TGG GGC CAT AGT GAG TGT G- 3´
CD163 - Rev in	5´ -TGG TGG ACT AAG TTC TCT CC- 3´
LILRA4 - Rev in	5´ -GTC TGC TGT AGG CTG TCA C- 3´
ZNF366 - For in	5´ -AGC TGA AGA GAC ACC TCA CC- 3´
ZNF366 - Rev in	5´ -AAC TCC CGC CCA CAA ATC C- 3´
CD1A - For in	5´ -TTC TAC CCA AAG CCC GTG TG- 3´
CD1A - Rev in	5´ -CAC CGC CAA GAT GAT GAA GC- 3´
DES - For in	5´ -GGC TAC CAG GAC AAC ATT GC- 3´
DES - Rev in	5´ -ACC GTC TTC TTG GTA TGG AC- 3´
MCAM - For in	5´ -CTC ACA CCA GAC TCC AAC AC- 3´
MCAM - Rev in	5´ -GGG GTA GCG TGA TCT CCT G- 3´
CSPG4 - For in	5´ -AGA AGC AAG TGC TCC TCT CG- 3´
CSPG4 - Rev in	5´ -GGG GCA TCT CAT GCT CAT AC- 3´
CD3D - For in	5´ -CAG CAT CAC ATG GGT AGA GG- 3´
CD3D - Rev in	5´ -AGG AGC AGA GTG GCA ATG AC- 3´
CD28 - For in	5´ -GGC ATC CCT TCA CAA AGG AC- 3´
CD4 - For in	5´ -GGG ATA CAG TGG AAC TGA CC- 3´
CD4 - Rev in	5´ -CTT CTG GTC CTC CAC TTC AC- 3´

CD19 - For in	5´ -CTG CCT GTG TTC CCT TGT G- 3´
MS4A1 - For in	5´ -CCA CTC TTC AGG AGG ATG TC- 3´
MS4A1 - Rev in	5´ -CTG GAG TTT TTC TCC GTT GC- 3´
CD22 - For in	5´ -GGA CTC TGT GAC CAT GAC C- 3´
CD22 - Rev in	5´ -AGG ATC TGA ACC GTG GAA GG- 3´
KLRB1 - For in	5´ -GCT GTG CTG GGA TTA TTC TC- 3´
KLRB1 - Rev in	5´ -CAG CAG GCT GGA TTC TTT GG- 3´
CD244 - For in	5´ -ACC TGC AAT GTC AGC AAT CC- 3´
CD244 - Rev in	5´ -CCT GGT TTT CAG ATC CTT GAC- 3´
NCR1 - For in	5´ -GAG CCC CAT TTC ATG GTT CC- 3´
NCR1 - Rev in	5´ -TTC TGT TAC CAC CAG ATC CAG- 3´
CD1C - For in	5´ -GTA TGT ACA CAG GCA AGT GAG- 3´
CD1C - Rev in	5´ -GTC TCA CTC GAC AAG ACA GG- 3´
CLEC4C - For in	5´ -ACT GGG ATG CAA TCT TGG AC- 3´
CLEC4C - Rev in	5´ -TCGCACAACGCTCATCAAGG- 3´
FCGR3A - For in	5´ -GGG TGT TCA AGG AGG AAG AC- 3´
FCGR3A - Rev in	5´ -GAT GAG ATG GTT GAC ACT GC- 3´
FCGR1A - For in	5´ -AAT CTG GTC ACC CTG AGC TG- 3´
FCGR1A - Rev in	5´ -TGA AAC CAG ACA GGA GTT GG- 3´
CD14 - For in	5´ -GGC AGG AGA TCA ACA TAA ACC- 3´
CD14 - Rev in	5´ -ACA AGG TTC TGG CGT GGT C- 3´
MRC1 - For in	5´ -ATT TGC GTG GCT GCA GAT GG- 3´
MRC1 - Rev in	5´ -CAG TAG CAG GGA TTT CAT CTG- 3´
MSR1 - For in	5´ -GAG GAC TCC CAG GAT ATG C- 3´
MSR1 - Rev in	5´ -GCT TGA ACA CCT GGG TAT CC- 3´
CLEC4A - Rev in	5´ -CAT TGC CAA TGT CGC TGA CC- 3´
CEACAM8 - For in	5´ -CAG TCA CCC TGA ATG TCC TC- 3´
CEACAM8 - Rev in	5´ -CTG ACT GTG GTC CTG TTG C- 3´
AZU1 - For in	5´ -GTT CCT GGC CTC CAT TCA G- 3´
AZU1 - Rev in	5´ -AGC AGC ATC AGG TCG TTC AG- 3´
ELANE - For in	5´ -CAC CAT CAA CGC CAA CGT G- 3´
ELANE - Rev in	5´ -GAT TAG CCC GTT GCA GAC C- 3´
KLRF1 - For in	5´ -GAG ATG AAA AGC TGG AGT GAC- 3´
KLRF1 - Rev in	5´ -TTC CTT AAT GGC AGC ACA GC- 3´
